# Mycobacteriosis and Tuberculosis: Laboratory Diagnosis

**DOI:** 10.2174/1874285801812010041

**Published:** 2018-03-30

**Authors:** Davood Azadi, Tahereh Motallebirad, Kazem Ghaffari, Hasan Shojaei

**Affiliations:** 1 Department of Laboratory Sciences, Khomein University of Medical Sciences, Khomein, Iran; 2 Department of Microbiology, School of Medicine, Isfahan University of Medical Sciences, Isfahan Iran

**Keywords:** Mycobacteriosis, Mycobacteria, Mycobacterial disease, Acid-fast staining, Laboratory diagnosis, Mycobacterium bovis

## Abstract

**Background:**

Tuberculosis is one of the most important infectious diseases that has claimed its victims throughout much of known human history. With Koch's discovery of the tubercle bacillus as the etiologic agent of the disease, his sanitary and hygienic measures, which were based on his discovery and the development of a vaccine against tuberculosis by Albert Calmette and Camille Guérin in 1921, an attenuated *Mycobacterium bovis* strain, bacilli Calmette-Guérin (BCG), and the discovery of the first antibiotic against tuberculosis, streptomycin by Selman Waksman in 1943, soon led to the opinion that appropriate control measures had become available for tuberculosis and it had been assumed that the disease could ultimately be eradicated.

The emergence of resistant strains of this bacteria and widespread distribution of the disease in the world, and the emergence of the AIDS epidemic destroyed any possibility of global control of tuberculosis in the foreseeable future.

**Objectives:**

The purpose of this review is to highlight the current scientific literature on mycobacterial infections and provide an overview on the laboratory diagnosis of tuberculosis and non-tuberculosis infections based on conventional phenotypic and modern molecular assays.

**Method:**

In this study, a number of 65 papers comprising 20 reviews, 9 case reports, and 36 original research in association with mycobacteriosis and the laboratory diagnosis of mycobacterial infections, were reviewed.

**Results:**

Based on our analysis on the published documents methods applied for the laboratory diagnosis of tuberculosis are continually assessed and developed in order to achieve more rapid, less expensive, and accurate results. Acid-fast staining and culture for mycobacteria remain at the core of any diagnostic algorithm with the sensitivity of 20-70% and specificity of 95-98% for AFB microscopy and the sensitivity of 95% and the specificity of 98% for culture based diagnosis. Following growth in culture, molecular tests such as nucleic acid hybridization probes and DNA sequencing may be used for definitive species identification. Nucleic acid amplification methods provide the means for direct detection of *Mycobacterium tuberculosis* in respiratory specimens without the prerequisite to isolate or culture the organism, leading to more rapid diagnosis and better patient care.

**Conclusion:**

As the researchers in a developing country, we strongly believe that despite significant advances in laboratory capacity, in many countries reliable confirmation of suspected mycobacterial diseases is hindered by a lack of knowledge on proper standardized methods, sufficient funds, suitably trained staff and laboratory supplies.

## 1 INTRODUCTION

### 1.1 Tuberculosis and Mycobacteriosis

Tuberculosis (TB) is considered one of the most important infectious diseases through the course of human history which can affect nearly any organ in the body, but it mostly causes lung infections. Historically, tuberculosis rose and fell repeatedly in the form of vast epidemics. After many unsuccessful attempts to control tuberculosis, it was finally controlled in the late 19^th^ century. The time was nearly synchronous to the identification and description of *M. tuberculosis* by Dr. Robert Koch and discovery of Bacillus Calmette–Guérin (BCG) vaccine by Albert Calmette and Camille Guérin [1].

In 1982, nearly any medical practitioners believed that tuberculosis would be eradicated before 2000 and its importance will only be confined to studying the history of medicine. But 10 years later, in 1993, these optimisms were no more than illusory when WHO declared TB a “Global Health Emergency” [2]. It has been estimated that about a third of world’s population are infected by TB sub-clinically. The occurrence of nearly 10 million new TB cases which only two third were “half cured” is more than evident for demonstration of the treat TB poses to healthcare system. According to recent WHO reports, about 8.8 million people fell ill with TB annually. The global incidence rate is 140 new cases per 100,000 and mortality rate is about 28 per 100,000 [3]. In Iran, according to the 2010-11 report of Ministry of Health’s TB and Leprosy Control Department, the incidence rate is 10-24 cases per 100,000 and 16% to 20% of cases are related to foreigners especially Afghans. The mortality rate is declared to be 7.1% in Iranians and 2.7% in foreigners [4]. In Iran, It has been a well-known disease since a very long time ago. It was, wrongly indeed, assumed to be caused by being cursed or depressed [2]. Islamic scientist’s al-Razi and al-Tabari described tuberculosis in their works [5].

In Iran, It has been a well-known disease since a very long time ago. It was, wrongly indeed, assumed to be caused by being cursed or depressed [2]. Islamic scientist’s al-Razi and al-Tabari described tuberculosis in their works [5].

Despite the discovery of effective vaccines and drugs for TB, about a third of world and a half of world’s immigratory population are still infected with TB. Although in recent years, the incidence rates dropped significantly, TB is still considered to be a global problem [2]. In order to be sure about this reduction as the product of better disease control, not as the result of wrong case reports, there should be a well-structured and organized system to arrange and identify all the disease cases. [2]

Of 175 mycobacteria species currently recognized, slow-growing mycobacteria are the most suspected pathogens for humans. Quite the contrary, most rapidly-growing mycobacteria are thought to be incapable of infecting humans [6, 7]. Today, with the emergence of immunodeficiency diseases as the product of HIV, cancers and out of control use of immunosuppresor drugs, opportunistic micro-organisms like Non-Tuberculous Mycobacterium (NTMs) are becoming prevalent pathogens in these patients [8, 9]. Nontuberculous Mycobacteria (NTM) are ubiquitous in the environment and cause colonization, infection, and pseudo-outbreaks in health care settings. Data suggest that the frequency of nosocomial outbreaks due to NTM may be increasing.

NTMs can cause certain conditions like pneumonia, lung abscess, pleural infection, meningitis, lymphadenitis and many infections of skin and soft tissue. These bacteria are all acid fast and are not much differentiated from *M. tuberculosis* using primary staining and microscopy preparation procedures. So, practically it is really hard to identify NTMs, their environmental resource and population and their persistence in ecosystems. So differential diagnosis of NTMs from *M. tuberculosis* and subsequently, the control and treatment of the underlying disease is hardly straightforward. As a result, one of the most important roles of modern microbiology laboratories is the diagnosis and identification of NTMs and differentiating them from very similar *M. tuberculosis* strains [10].

## 2 METHODS AND MATERIALS

Increasing evidences are pointing to the hospitals’ soil and water storages as the source of NTMs that are frequently transmitted to patients [11, 12]. Additionally, the presence of atypical mycobacteria in the periphery regions of hospitals makes them another suspicious source of Hospital-Acquired Infections [HAIs] and reduced hot water temperatures may be partly responsible for this phenomenon.

The media for such transmissions includes [11-14]:

_*_ Intra Venus Drugs
_*_ Water Purification Filters
_*_ Bronchoscopy Devices
_*_ Prosthetic Heart Valves
_*_ Infected Intra-Abdominal Shunts [result in peritoneal infection]
_*_ Infected Aerosol Particles in Ventilation Systems [result in lung infections]
_*_ Dialysis Devices

### 2.1 Laboratory Diagnosis of TB

The role of laboratory have always been important in the diagnosis and treatment of TB. There are many laboratory procedures concerned with the diagnosis of TB including: isolation of microbial agent, identification of the causative species, and determining drug sensitivity of the isolates. In the new millennium, the success of control programs for TB is directly dependent on the diagnostic ability of the laboratory network. In developed countries, using new technologies has made diagnosis, identification and drug sensitivity tests fast and reliable. These technologies are working in conjunction with the suitable health plans [15]. On the contrary, many developing countries that have high TB incidence, are in the pursuit of new microscopic and cultural approaches that are seldom available in these countries. For a long time, effective control of TB was easily achieved by using microscopic diagnostic tests in conjunction with very organized treatment plans. But, ineffective management and the inadequate aid of TB control and laboratory diagnosis programs, stopped the progress of management of control of this life-threatening disease. Additionally, complications due to epidemics of HIV and MDR (Multi Drugs Resistant)-TB especially in Africa, Asia and East Europe are also at play in stopping the progress of TB management. Because in these regions effective management and control of TB is totally dependent on microscopic observations. Effective control includes accessibility of laboratory services at any level which practicability is dependent on the management and support of non-concentric laboratory networks, providing suitable and reliable TB diagnostic and control services, in different areas. Although reinforcement of laboratories is itself dependent on statement of higher diagnosis and identification priorities, as reflected on the new strategy on TB control, there should be higher effort to improve accessibility and use of current diagnostic procedures and also development and Implementation of new diagnostic technologies [16, 17].

New technologies like Fluorescent Microscopy [FM], liquid cultures for identification and drug sensitivity tests and reinforced procedures to identify and study drug resistance needed much concentrated work and is being developed only gradually. Today, many studies are in process in order to introduce modern diagnostic procedures for developing countries. The basic strategy of FIND [Foundation for Innovative New Diagnostics] is using organized, well-planed R&D studies to achieve new diagnostic and identification technologies [18]. Although, the studies are concerned with economic aspects, usability and achieving new diagnostic approaches, most of them ignore the need to well-educated team, quality management systems and other pre-requirements and practical standards. As long as medical practitioners distrust the reliability of laboratory procedures, they will continue treating patients using their own experience [19]. These setbacks are motivating the efforts to reinforce laboratory system in parallel with efforts to development and implementation of new techniques and approaches.

The complications of MDR TB and the recent incidence of (Extensively drug-resistant) XDR TB are sufficient motives for enhancing *M. tuberculosis* identification techniques and drug sensitivity tests [20]. The complexities faced in the way of providing accessible services, are by themselves further complicating the discussion around management, analysis, education, HRM [human resources management] and developing laboratory networks. For the countries that TB laboratory services are integrated with general laboratory services or is maintained as a big privately held segment, the question is “Will the national TB control problem can successfully improve the quality and accessibility of laboratory services or more generally, just increase their capacity. Evidences showing that previous efforts for offering a separate and parallel microscopic TB laboratories is not so appropriate to enhance health care system. Today, the quality of TB laboratories is improving and this is acting as a catalyst or on the contrary the limiting factor of other TB control segments. This article intends to review current outlines for the key technical and organizational challenges related to direct microscopy, culture and, drug sensitivity tests, and molecular techniques of diagnosing TB disease.

### 2.2 Microscopic Diagnosis

In developing countries, which their facilities and equipment’s seldom satisfy the needs for suitable culturing, obtaining smears and microscopic observation is their primary laboratory procedure for the diagnosis of TB. Although the sensitivity of acid-fast staining is less than culturing procedures and its sensitivity is also dependent on the technician skill and the use of appropriate approaches, but it is the fastest and the most essential TB diagnosis tool especially for patients whom microbial burden and the risk of transmission are very high or the patients who need rapid onset of therapy [21, 22].

According to 2013 report of WHO, the sensitivity of the identification of pulmonary TB using microscopic smear examination is estimated to be up to 70% [4]. The obvious thing is although there are many diagnostic approaches for TB, WHO still considers microscopic smearing of phlegm as the primary diagnostic approach. So the diagnosis of pulmonary TB is usually made based on clinical symptoms, chest radiography and the result of microscopic smear tests.

The sensitivity of phlegm smear test with Ziehl–Neelsen stain in different studies has been estimated between 20% and 70% requiring at least 10^5^ bacilli in each ml of phlegm for test to become positive [23]. To increase the sensitivity we need to increase the quality of materials, tests and the skill of team to a whole new level.

The simultaneous epidemics of HIV and TB, especially in developing countries and the fact that Ziehl–Neelsen stain is less sensitive in people with HIV infection, caused many studies to be concentrated on enhancing microscopic identification approaches. In developed countries, concentrated smears with FM staining is usually the primary approach. This combination is highly effective for low income countries which have high HIV incidence rates but it needs more funding [24]. EQA (External quality assessment) programs are also needed in order to make sure the sample preparation and result interpretation procedures are well implemented in all laboratory units. For EQA programs to be implemented, we need well educated and specialized co-workers for supervision on the laboratory environment and proof reading the results from every laboratory. Although in order to implement evaluation programs in a given country, region or city, international instructions recommend random choice of one or more smears and using these samples as the criteria for EQA programs in the level of that region [24, 25].

Implementing EQA for microscopic examinations not only reinforce laboratory networks, but it improves the quality of diagnostic procedures for TB in the underlying region.

Systematic evaluation of control programs and result interpretation by a laboratory specialist is the most important component for enhancing the management of TB laboratory networks. But due to the lack of enough time and resources, such evaluations are usually very limited. This approach leads to more efficient and cost-effective use of human resources and shows an appropriate management program in the countries with limited resources.

### 2.3 Mycobacterial Culture in the Diagnosis of TB

The chance of appearance of acid-fast bacillus in the stained sample is directly proportional to the number of bacilli in the patient’s phlegm and if that number be less than 1000 per ml, the chance of finding acid-fast bacillus would be less than 10% [26].

In culturing approach, the minimum number of bacilli in phlegm can be as low as 100 for diagnosis to become positive which in comparison to the microscopic tests, has far more sensitivity. Additionally, with culturing approach, the exact species of mycobacterium can be detected using complementary biochemical and other diagnostic tests [26].

However, using the microscopic smear approach, differentiating pathogenic and non-pathogenic mycobacteria is seldom possible because both groups are acid-fast and morphologically similar. In addition, the specificity of *M. tuberculosis* culturing tests is decreasing due to possibility of contamination and technical errors causing false positive results [26].

According to WHO standards for the diagnosis of pulmonary TB, acid-fast smears are first obtained from patient’s phlegm and for negative results, specific culturing environments will be used as the golden standard. But results from various studies demonstrate that depending on the stage of the TB and the presence of bacilli in the sample as well as geographic location, sensitivity and specificity of acid-fast tests are estimated to be between 73-95% and 95-98%, respectively. Also, the chance of occurrence of false positive results is about 1.6-4.7%. So in countries with high incidence of TB, the specificity of smear tests are higher. But in low incidence countries, the value of culturing and other approaches for the identification and differentiation of TB from other TB-Like diseases brought by atypical mycobacteria is undoubtable [27].

Considering the high costs and instruments needed to perform mycobacteria culturing tests, there are many debates going on about using this approach in high incidence regions, like developing countries in order to identify TB patients [28]. However, many countries with high burden of infections don’t even have the most basic instruments for developing exact and reliable culturing approaches for performing drug sensitivity tests and diagnosis of MDR-TB. This, in turn, makes the data about drug-resistance strains in these regions inaccessible [28]. Additionally, national TB control programs must promote appropriate use of current limited culturing capacities in order to make this limited resources accessible for the whole countries. So, priorities should be determined [29].

## 3 PHENOTYPIC APPROACHES FOR IDENTIFICATION OF MYCOBACTERIA

### 3.1 Pigment Production Test

Mycobacteria, dependent on the species, are able to produce carotenoid pigments with different types and amounts. On this basis, mycobacteria are classified into three groups including: Photochromogenic [producing pigments in the light], Scotochromogenic [producing pigments in the dark], and non-chromogenic. With pigment production test we can classify the isolates in one of the above mentioned groups [30, 31].

### 3.2 Growth Rate Calculation

Generally, mycobacteria are divided into two groups based on their growth rate: slow growing and rapidly growing. In order to calculate growth rates of mycobacteria, a special culture medium named N-Medium is used and is placed in incubators for as long as 18 days. As a rule of thumb, slow-growing mycobacteria cause turbidity in culture medium in more than 7 days, while rapidly growing strains do the same in less than 7 days [30, 31].

### 3.3 Niacin Reduction Test

Niacin is one of the B group vitamins that is produced by all mycobacteria. But some mycobacteria including *M. simiae* and *M. tuberculosis* lack the enzyme for conversion of niacin to Ribonucleotides. On this basis, the accumulation of niacin in the culture medium is important for the identification of these species. However this test would be negative for all other mycobacteria [30, 31].

### 3.4 Nitrate Reduction Test

Some mycobacteria, especially *M. tuberculosis* are able to reduce nitrate to nitrite. The nitrate reduction test is performed for the diagnosis of *M. tuberculosis, M. kansasii*, *M. szulgai* and also some non-pathogenic photochromic stains. *M. fortuitum* is also nitrate positive. *M. avium, M. xenopi, M. simiae and M .marinum* are all nitrate negative or hardly positive [30, 31].

### 3.5 Catalase Test

Catalase is a soluble intracellular enzyme which breaks hydrogen peroxide into water and oxygen. The presence of oxygen bubbles in the mixture is the criteria for this test to become positive. Mycobacteria have various catalyze enzymes including:

### 3.6 Semi-quantitative Catalase Test

The semi- quantitative catalase test is valuable in separation of some mycobacterial species. *M. gastri, M. avium, M. marnium, M. tuberculosis, M. haemophilum* and *M. bovis* produce a bubble column with a height less than 45 mm while the other species make higher columns.

### 3.7 68 degree Celsius Catalase Test

The test is positive for only a few of mycobacteria which their catalase enzyme can resist to being heated at 68 degree Celsius for 20 minutes [30, 31].

### 3.8 Iron Uptake Test

This test is only positive for rapidly growing mycobacteria like *M. furtitum* and *M. fline* that can absorb iron salts form the culture environment [30, 31].

### 3.9 Potassium Tellurite Reduction Test

Reduction of colorless potassium tellurite to metallic black tellurite in the course of 3 to 4 days, is a determinant of presence of *M. avium* complex. This test is also positive for rapidly growing mycobacteria [30, 31].

### 3.10 Tween 80 Hydrolysis Test

Enzymatic hydrolysis of tween 80 by mycobacteria results in the degradation of this compound. As the result, complexed neutral red is released and the color of the test substrate will change. This color change is due to hydrolysis of the tween. This test is useful in the diagnosis of scotochromogenic and non-chromogenic mycobacteria [31, 32].

### 3.11 Arylsulfatase Test

Arylsulfatase is an enzyme that hydrolyses the bound between sulfate and aromatic circle in the compounds with R-OSO3H formulae. If phenolphthalein 3-potassium disulfide substrate be available in the culture medium, arylsulfatase hydrolyses this compound and releases phenolphthalein which will become red in basic environments. Determining arylsulfatase enzyme activity is useful in differentiating fast growing mycobacteria from non-Photochromogenic ones [30, 31].

The details of biochemical properties of some clinically important Mycobacterium are presented in Table **1** and Fig. (**1**).

## 4 MOLECULAR APPROACHES FOR TB DIAGNOSIS

For rapid TB diagnosis, there are various molecular diagnosis approaches available that work on the basis of targeting and multiplication of different areas in mycobacterial DNA [32]. Nucleic-acid amplification tests (NAATs) targeting *Mycobacterium tuberculosis* (MTB) have enormous potential to improve TB case detection, with commercial NAATs, such as the GenProbe Amplified® *Mycobacterium Tuberculosis* Direct (MTD) Test (Gen-Probe Inc, San Diego, CA), reported to have nearly perfect sensitivity in sputum smear-positive patients and a sensitivity of 61–76% in smear-negative patients. Although false positive results seldom occur, but they can occur as the product of contamination of sample with environmental mycobacteria and unspecific attachment to the probe. But in comparison, false negative results are more common and it is usually occurs because of the low number of mycobacteria in the CSF samples or because of the limiting factors opposition molecular interactions [32].

Delays in adopting NAATs for TB reflect ongoing operational and feasibility concerns in low-income settings [26], but a lack of information about the clinical impact and incremental value of these tests beyond the standard diagnostic algorithm of sputum smear microscopy and clinician judgment may also have contributed to the slow uptake of this technology. In a prospective diagnostic cohort study of patients suspected of TB in a low-income country with a high prevalence of TB and HIV, it has been shown that NAATs identify many smear-negative TB patients whom clinicians would otherwise fail to diagnose. Although the sensitivities of the NAATs evaluated in several studies were modest compared to mycobacterial culture, it has been found that, if routinely applied, same day NAAT would have decreased time-to-treatment initiation in smear-negative TB patients by almost four weeks [26].

### 4.1 Nucleic Acid Hybridization Techniques

The use of nucleic acid hybridization techniques allows rapid identification of certain common mycobacterial species. Commercially available nucleic acid probes are available for *M. tuberculosis* complex, *M. avium* complex, *M. kansasii* and *M. gordonae*. These tests used with nonisotopically labelled (acridine ester- labeled nucleic acid) probes specific for mycobacterial ribosomal RNA (rRNA). The RNA is released from cell after sonication. The DNA probe is allowed to react with the solution. If specific RNA is present, a stable DNA, RNA complex, or hybrid is formed. The complex is detected by alkaline hydrogen peroxide solution. The hybrid bound acridine ester is available to cause a chemiluminescent reaction, resulting in the emission. The amount of light is emitted of light is related to the amount of hybridized probe [33, 34].

### 4.2 Direct Nucleic Acid Amplification Test

Nucleic acid amplification assay designed to detect mycobacterial species directly from patient’s specimen can be performed in a few hours on processed specimens and offer the promise same day reporting of the result. The amplified *Mycobacterium* direct test (AMD) consist of transcription mediated amplification of a specific 16S rRNA target performed at a constant temperature for detection of *M. tuberculosis* and NTM in smear positive and negative specimens [33, 34].

### 4.3 Gene Sequence Analysis

Sequences of several genes are considered good targets for species identification, although it is usually necessary to analyze several sequences at a time. In particular, 16SrRNA, rpoB and Internal Transcribed Spacers (ITSs) have been shown to be useful for the rapid identification of many mycobacterial species [33, 34], including *M. malmoense*, *M. szulgai*, and *M. flavescens*, which are hardly identified with conventional methods [33, 34]. A list of major genes that might be used for differentiation between mycobacterial species is shown in Table **2**.

An important application of gene sequence analysis in mycobacterial diseases is the detection of drug resistance. It not only provides clinically relevant information but also assists in deciphering strain relatedness. Several genes of mycobacteria can be analyzed to detect drug resistance; some of them that overlap housekeeping genes are used for species identification [33, 35]. A list of major *M. tuberculosis* genes that have been linked with the acquisition of drug resistance is presented in Table **3**.

#### 4.3.1 PCR-RFLP

At first, analysis of gene sequences was addressed with PCR-restriction fragment length polymorphism (RFLP) analysis, otherwise called the PRA, or PCR-restriction enzyme analysis (REA), method. This method combines PCR amplification and restriction analysis. The pattern obtained after electrophoresis is species or strain specific [36].

##### 4.3.1.1 SNP Analysis

Two major lines of research based on SNP analysis include lineage specific typing and determination of the occurrence of mutations leading to drug resistance. SNPs exhibit low levels of homoplasy; however, convergent evolution, especially within drug susceptibility-related genes, is considered common [37].

The SNP at a particular location might be detected by REA [37] or by a variety of PCRs. Modern technology provides several efficient methods to analyze several SNP sites at a time. They can be addressed with molecular beacons, as they are able to distinguish sequences that differ by even a single nucleotide substitution [38]. Next, they can be detected by identifying shifts in melting temperatures obtained by real-time PCR curve analysis [39]

##### 4.3.1.2 Genome Analysis PFGE

The first molecular typing methods for *M. tuberculosis* genome were based on RFLP analysis of bacterial DNA. Here, chromosomal DNA isolated from different mycobacterial strains is digested by using various restriction enzymes. The resulting restriction fragments are separated by gel electrophoresis and visualized with UV light. The observed fingerprint patterns are strain specific [40].

#### 4.3.2 RAPD Analysis

Randomly amplified polymorphic DNA (RAPD) analysis or arbitrarily primed PCR (AP-PCR) is a typing method that has increasingly been used for estimating genetic variability among different bacterial taxon’s. This method requires no previous knowledge of the template DNA sequence. By using a single, arbitrarily designed primer with a length of 5 to 50 bp and low-stringency conditions, the primer anneals to template DNA at both perfectly and partially matched sites, resulting in strain-specific multiband DNA profiles [41].

##### 4.3.2.1 AFLP

AFLP analysis is a PCR-based method in which DNA is digested with two restriction enzymes, a rare cutter and a frequent cutter, which have 6- and 4-bp recognition sites, respectively. The resulting restriction fragments are ligated to double-strand adaptors (10 to 30 bp) recognized by PCR primers that are complementary to the adaptor sequence, carry the restriction site sequence, and contain selective bases at their 3= ends. The use of radiolabeled primers allows visualization of PCR products by means of autoradiography [42].

##### 4.3.2.2 Deletion Mapping

Deletions or, rather, LSPs can be used as molecular markers to study genetic variability among mycobacteria. The LSP-based methodology relies on previous knowledge of the analyzed sequences, and it usually requires relatively large quantities of DNA (micrograms) [43].

##### 4.3.2.3 Whole-genome Sequencing (WGS)

The completion of the genomic sequence of *M. tuberculosis* H37Rv [44] has commenced a whole new chapter in the epidemiological study of mycobacteria. The development of second-generation sequencing (SGS) and further-generation sequencing platforms has made studies on mycobacterial epidemiology as informative as they have never been before.

WGS provides information about the whole genome, and this method can identify virtually all varieties of markers detected by the above-mentioned genotyping methods. It is therefore much more accurate and precise in detecting variability among strains and provides a wealth of information at every level possible, from global (population), through local (community) and individual host (single patient), to pathogen (strain) itself [45].

##### 4.3.2.4 Spoligotyping

Clustered regularly interspaced short palindromic repeats (CRISPRs) comprise a family of widely encountered repetitive DNA elements. While initially detected in *Escherichia coli* [46], these elements have been subsequently identified in 40% of bacteria and 90% of archaea [46]. The CRISPR loci generally consist of a noncoding, A/T-rich leader sequence and variable numbers of identical direct repeats (DRs) interspersed with unique spacer sequences or spacers. Adjacent to CRISPRs are often CRISPR-associated (Cas) genes, together forming a CRISPR-Cas genomic region. CRISPR loci are thought to represent a sort of prokaryotic adaptive immunity system that confers resistance to phages [47]. The number of spacers within CRISPR loci is variable. Spacers may be acquired from a viral invader as a specific way of memorizing phage infection [47]. On the other hand, some spacers may be deleted as a result of transposition and homologous recombination between neighboring or distant DRs. After the incorporation of the spacer, the mechanism of phage resistance is conferred by the expression of this sequence, hybridization, and cleavage of foreign RNA or DNA [47].

CRISPR loci have been identified in several mycobacterial species [48]. However, long CRISPRs have been found in *M. tuberculosis*, *M. bovis*, and *M. avium*. As the integrated CRISPR Cas system can be found only in *M. tuberculosis* and *M. bovis* different systems in NTM are thought to have been acquired by horizontal gene transfer from other bacteria [48]. It is still unknown whether the CRISPR system is functional in mycobacteria. It seems that while it might interfere with incoming nucleic acids, it might have lost the capability of incorporating new spacers [48].

Spacer oligonucleotide typing (Spoligotyping) is a PCR-based technique for MTBC strain differentiation that takes advantage of the structure and polymorphism of the DR locus. In spoligotyping, the entire locus is amplified by PCR by using two inversely oriented primers complementary to the sequences of DRs. A biotinylated reverse primer is used so that all the reverse strands are labeled. Next, PCR products are hybridized to a membrane with a set of 43 immobilized, covalently bound, synthetic oligonucleotides, each representing a unique spacer identified by sequencing of the DR locus in *M. tuberculosis* H37Rv (spacers 1 to 19, 22 to 32, and 37 to 43) and *M. bovis* BCG vaccine strain P3 (spacers 20, 21, and 33 to 36). After hybridization, the membrane is incubated with a streptavidin-peroxidase or streptavidin-alkaline phosphatase conjugate, and the hybridization signals are detected by chemiluminescence. Strain-specific patterns (spoligotypes) are then visualized on X-ray film. Strains are differentiated by the presence or absence of individual spacers in the complete 43-spacer set [49]. Since spoligotyping results can be presented as a binary system (present/absent), they can be easily interpreted, digitized, and compared among different laboratories [49].

##### 4.3.2.5 Rep-PCR

Rep-PCR (DiversiLab system; bioMérieux, France) is a commercially available, high-throughput, automated system for typing of multiple Mycobacterium species based on the variability generated by repetitive sequences interspersed within the genome [50]. This procedure involves the amplification of repetitive, noncoding sequences and their separation using microfluidic electrophoresis over a chip. As the fragments migrate over the chip, their size and fluorescence intensity are measured by a laser, thereby generating a graph.

### 4.3.3 IS 6110-RFLP Analysis

 Study of the complete *M. tuberculosis* H37Rv reference strain genome sequence revealed a relatively large amount of repetitive DNA elements [51]. Those elements vary in length, structure, and localization. Two main groups can be distinguished, that is, Tandem Repeats (TR) and Interspersed Repeats (IR).The first are short monomeric sequences (up to 100 bp) organized as head-to-tail arrays, whereas the latter are scattered as individual copies throughout the entire genome. An important class of IR sequences is Insertion Sequences (IS), which are mobile genetic elements.

The best known and investigated insertion sequence is IS*6110* first recognized by Thierry *et al*. in the early 1990s [52]. Differences in the copy number and locations within the genome, responsible for the high degree of IS*6110* polymorphism, have predisposed this sequence to be used as a specific molecular marker for genotyping of *M. tuberculosis* strains [53].

The sensitivity and specificity of these tests are evaluable using microbial culturing test results. Although this evaluation is hardly easy specially when considering in 20-30% of pulmonary TB tests and even more portions of non-pulmonary tests, the results are negative. Approaches based on multiplication of surrogate nucleic acids can’t replace clinical diagnosis, acid-fast staining and culturing procedures in TB diagnosis but can’t be used as a complementary test beside them [54].

Generally, it can be concluded that nucleic acid multiplication tests can be used as a tool for TB diagnosis. But due to relatively low sensitivity, and high incidence of false-positive results, the negative results of these kinds of tests can’t be reason enough to totally eliminate the possibility of TB infection [32, 54]. The important point is that these tests can’t differentiate between alive and dead micro bacteria and thus may not be used to assess the efficiency of treatment. Finally, performing nucleic acid multiplication tests is absolutely dependent on enough laboratory experience [32].

Although the specificity of PCR molecular techniques can be very high but their sensitivity is low in comparison to culturing approaches which are considered to be the golden standard. However, if the quality of clinical specimens be high enough, PCR based approaches become hopefully more sensitive. In the very best conditions, assuming the test is performed in a modern laboratory with experienced technicians, the sensitivity of PCR tests is 90% for smear-positive and culturing-positive samples and 40-77% for smear-negative samples [32, 54].

PCR based tests can reduce the time needed for diagnosis of TB to less than 3-6 hours, but due to high costs and requirements, they are seldom the most straightforward tests in developing countries. However, in the systems in which performing culturing test are difficult, PCR can be used as an alternative approach [54].

## 5 IDENTIFICATION OF NTMS IN CLINICAL SPECIMENS

Differentiating clinical manifestation of NTMs from *M. tuberculosis* is very difficult. This makes exact identification of NTMs really important and thus different pathogenic NTMs in different regions should be separated and identified in order to manage and treat infected patients [55].

In developing countries like Iran, diagnosis of TB is usually dependent on microscopic tests because in such countries, culturing and drug-sensitivity test facilities are practically inaccessible. According to 2013 WHO report 70% of pulmonary TB cases in Iran are identified by microscopic smear tests. So, physicians may ignore the possibility of NTMs in smear positive results and thus appropriate treatment cannot be achieved. So appropriate diagnostic and differential methods must be implemented in TB diagnosis laboratories in order to differentiate NTMs from *M. tuberculosis* [56, 57].

The separation and identification of NTMs from clinical specimens is usually dependent on special preparation procedures including various anti-contamination and incubation techniques like using different temperatures or atmospheres containing 5-10% CO_2_ to promote growth rate. As these technologies are seldom available in most laboratories, NTM infections usually cannot be identified. On the other hand, phenotypic identification of various NTMs is dependent on at least 10 different tests; therefore, reliability of final result is usually hazy because of the difficulties faced in interpretation of various tests as well as the low sensitivity [57, 58].

Thus, fast and reliable diagnostic techniques for identification of NTMs and exact mycobacterial species need to be developed. These techniques include various molecular tests like molecular probes, *hsp*65 gene based algorithms, 16S rRNA sequencing techniques and non-molecular tests conjoined with key phenotypic tests [59-61].

## 6 DRUG SUSCEPTIBILITY TESTS FOR THE ASSESSMENT OF DRUG SENSITIVITY AND RESISTANCE

Nowadays, despite of passing more than 70 years of discovery of the first anti-tuberculosis antibiotic [streptomycin in 1943] and the other postdated suggested drugs, tuberculosis has been a serious issue and one the most important reasons of human beings' mortality yet [62, 63].

The known reasons of crescent resistance of mycobacterium tuberculosis to drugs can be: not applying the appropriate remedy (improper drug prescription, using one-drug diet instead of multi-drug remedies, interruption in drug appliance), lack of appropriate control of tuberculosis in some countries, decrease in effectiveness of anti-tuberculosis drugs and finally prevalence of HIV [64].

The treatment of infections caused by TB and NTM is generally obtained by surgery, drug appliance or both of them. Drug treatment of these diseases is quite costly, protracting and very often followed by consequences of drug toxicity. In some cases infected by resistant strains of mycobacterium tuberculosis, first and second generations of therapeutic diets are not effective. On the other side the difference of therapeutic diets used for sundry strains of NTM, and for slow-growing and fast-growing species seek antibiotic susceptibility tests. The suggested remedy for most of the slow-growing species consists of rifampin, Ethambutol and macrolide antibiotics for 18-24 months; in more complicated cases the necessity of drug susceptibility tests would be come up. For fast-growing species the remedy is often chosen according to drug susceptibility tests. In rarer cases ex. (*M. abscessus*) steady therapeutic diets consist of macrolide amikacin and tigecycline are preferred [65]. In order to determine the microbial susceptibility of mycobacterium species different methods are sought such as:

### 6.1 Absolute Concentration Method

In this method, the species of interest should be standardly inoculated in mediums of different drug concentrations. One of these mentioned concentrations is the critical concentration which is useful for determining the Minimum Inhibitory Concentration (MIC). The growth in critical and higher concentrations indicates the resistance of the mentioned specie. This growth on antibiotic containing mediums then is compared with drug free mediums with the intention of controlling [66, 67].

### 6.2 Inhibition Ratio Method

This method is similar to absolute concentration method but the MIC is determined according to standard strain H37Rv of mycobacterium. Subsequently, the result is the ratio of drug MIC which doesn't inhibit the growth to MIC needed to inhibit the growth of the standard strain H37Rv. This method is more commonly used for *M. tuberculosis* strains in contrast to other mycobacterium species [66, 67].

### 6.3 Proportion Method

This method which is still suggested by WHO for testing the second-line therapy drugs includes the usage of drug containing mediums that two prepared standard concentrations of bacteria are sequentially fecundated in mediums containing the drug and not containing it. The number of colonies formed in drug containing mediums is calculated according to the most dilute inoculation and then is compared to the number of colonies formed in drug free mediums of the same concentration. If the ratio of bacilli grown on the drug containing medium to the non-containing ones was more than %1, the mentioned specie would be resistant to the used drug [66, 67].

### 6.4 Disk Diffusion Method

In this method a disk with a certain amount of anti-microbes is placed on the solid medium which the examined bacteria is cultured on it. The size of the zone of inhibition around the disk is measures and then is compared with the scales determined by CLSI. This technique is chiefly used to determine the microbial susceptibility of fast-growing species, but concerning the slow-growing species the disk diffusion is not recommended because of different growth rate, lack of appropriate culture mediums, long term keeping and also the possibility of pollution during the incubation [66, 67].

### 6.5 Disk Elution Method

In this method, the disks containing a certain amount of antibiotic are added to liquid Oleic Albumin Dextrose Catalase (OADC) growth supplement and then are added to the melted medium. The next step is mixing the contents and then divide them among the plates of growth medium; the plates have to remain in room temperature in order that a solid state is obtained. The results of growing or not growing are reported according to the amount of antibiotic that each disk contains [68].

### 6.6 Broth Macro-Dilution Method

This method was used in 1970 for the first time in order to determine the antibiotic susceptibility of *M. Tuberculosis*. To determine the MIC using broth macro-dilution method, a certain concentration of the target microbial specie have to be fecundated in liquid state mediums possessing a series of concentrations of the antibiotic. The control of the experiment is the vial which has no antibiotic inside. In this method, the MIC is defined as the lowest concentration of antibiotic which no signs of growth are observed in. The discussing method is same as the proportion method and sometimes itself is called the proportion in liquid state if matter [69].

In order to accelerate this whole process the “Bactec broth macro-dilution method” is used, which comprises the measurement of C14 that is formed during the oxidation of the 3H-uracil of a ribonucleic acid [69]. Nowadays, the BacTec460 system is completely abolished, and instead of it the Mycobacterial Growth Indicator Tube (MGIT) is used in order to determine the antibiotic susceptibility of M. Tuberculosis strains and mycobacterium species [70, 71].

### 6.7 Broth Micro-Dilution Method

Broth micro-dilution has been stated as the golden standard with the intention of drug resistance test in bacteriology after 1971 [72]. In 1982 the first report published about the application of this method for testing the drug susceptibility of *Mycobacterium* [73]. The mentioned method is chiefly similar to broth macro-dilution method, but it is performed in 96-well plates which a series of antibiotic concentrations is inserted in them. Then every well is filled with 100 uL of a suspension formed of the bacteria of interest in a broth medium with a concentration about 5-10*5. The control well is only filled with bacteria suspension. In this experiment, the MIC is defined as the lowest concentration of antibiotic which bacteria does not grow in it [72, 73].

### 6.8 Epsilon Tests

This test was used for the first time in 1990 in order to determine the Mycobacterial antibiotic resistant. Determining the MIC using this method begins with the appliance of plastic strips impregnated with decreasing antibiotic concentrations. Numbering the MIC is similar to proportion method or certain concentration method. Using two antibiotic for one strain at the same time is one the advantages of this method [74].

## 7 ROLE OF HUMAN RESOURCES IN TUBERCULOSIS DIAGNOSIS

The staff of microscopic and source laboratory networks really seek expert lab scientists out in order to perform such subtle experiments. The lack of sufficient human resources limits the activity of laboratory networks in many aspects and in many countries. So that in a country with limited human resources [ex. developing countries] the shortage of lab technicians implies the governments to instruct new staff which they have no noticeable higher degrees. Such practical individuals who they are trained in a working environment can be very useful for diagnosis of Tuberculosis and also expeditious diagnosis of HIV; but they should be trained and supported in different levels and also are supposed to perform EQA to monitor their daily activity [75].

Academic instructions to these technicians lasts about 2-3 years in different countries, most of these instructions are about increasing the skill of culture and Developmental systems theory (DST) for various organisms and these courses themselves limit the acquisition rate of specific experiments in specific fields like Tuberculosis. Therefore, the need of instruction schedules with the intention of diagnostic skills is more necessary for graduated students of higher education. One of the most significant deficiencies in human resources is about the lack of specified goals and schedules for leaders and managers of laboratories. While in countries with an adequate human resources the management of a laboratory is handled by expert related doctorates, in developing countries the running of a specialized laboratory rests on the shoulders of normal educated people. Besides most of the doctorates held to investigative projects and despite of their higher education they are not involved in improving the health of the society. As a conclusion, a serious revision about the policy of management and instruction of new technologies and activities is needed in order to bring expert managers up.

## 8 LABORATORY SAFETY

One of the necessities of every step of diagnosing the M. Tuberculosis is the safety and health of all the staff who they are in touch with tuberculosis containing mediums. National reference laboratories [NRL] have to communicate the safety instructions to the diagnostic laboratories; and this task is a part of the national control project of Tuberculosis. In order to attain this goal, these schedules have to be presented by a combination of safety and laboratory instructions to help the promotion of danger assessment of different lab methods and to find the safest method. The advancement of laboratory equipment’s, tools and supplies can itself enhance the safety level.

The proper air conditioner, where the smears are prepared and microscopic observations happen, can really decrease the risk of infection in such areas [76]. So that a simple cabinet or air conditioner can remarkably decrease the expenses and also improve the safety level in contrast to safety biologic cabinets which are followed by other complications [76].

In order to control Tuberculosis in developing countries the culture capacity and DST have to be advanced, and this topic can be achieved by maintaining the least safety standards. The risk of these infections is noticeably high for those who are performing culture, diagnostic and susceptibility experiments. This risk challenges the mentioned countries to provide sufficient equipment’s and safety instructions and also to use suitable biologic safety cabinets in order to guarantee the employees' health.

## 9 QUALITY SYSTEMS

The limited quality of laboratory services for diagnosing Tuberculosis is the challenging barrier against microscopic experiments, DST culturing and NAAT methods. Many countries are still trying to start an efficient EQA to exploit it in all public and private diagnostic centers. The importance of an efficient EQA is more prominent when Tuberculosis cases are diagnosed with a HIV infection because the available cases has a few bacillus, so it is up to the laboratory to diagnose tuberculosis in such cases. In the meantime, EQA can provide a suitable practical approach with the intention of improvement and acceleration of susceptibility tests for these centers [77].

EQA is a part of quality systems of laboratories and a confirmed schedule for evaluating microscopic experiments and DST [78]. One of the most efficient methods suggested by NRLs is using international reference laboratories and interchanging strains between them in order to evaluate the function of most laboratories and of course inspecting global drug resistance. Considering the culturing has more difficulties and EQA schedules can not specify the exact susceptibility of culturing method in diagnosing Tuberculosis. The low efficiency of culturing method has been appeared in some drug resistance controlling approaches *i.e*. some laboratories have had difficulties in diagnosing tuberculosis from a positive sputum sample.

These quality problems cannot be solely solved by EQA, and all the involved factors such as documents, records, staff, standards and all the equipment’s have to be considered by all of quality management systems. In the meantime, a distinct feature of the developed countries is the existence of laboratory provisions and also credential plans for different kinds of diagnostic methods. When the laboratory standards are ascertained, performed and also controlled by countries, the NRLs can achieve all the mentioned goals mentioned before.

## 10 DISCUSSION

Nowadays, the Multi-Drug Resistant (MDR) and Extensively Drug-resistant (XDR) tuberculosis is not limited to developing countries anymore. People and diseases are continually on the move. Besides, about one third of the world population are infected by Mycobacterium tuberculosis unawares [79]. These people infected concealed are rolling as a storehouse for the next manifestation in the future. So this fact that all the mycobacteriology laboratories have to able to diagnose mycobacterium tuberculosis has a high level of importance.

Tuberculosis is still one of the health difficulties of Iran and also the world and put thousands of people to death daily. The main reasons which really challenges the diagnosis of tuberculosis are: lack of a comprehensive management system for the tuberculosis diagnostic laboratories, the paucity of quality control systems and quality guarantee systems for the tuberculosis diagnostic experiments, the sluggish process of diagnostic experiments like sputum culturing and other samples which need 4-12 weeks of time, the usage of an antiquated method as the only diagnostic method, insufficient knowledge of laboratory staff, appearance of non-tuberculosis mycobacteria acting as an opportunist and is the cause of infections very similar to tuberculosis. The mentioned tuberculosis mycobacteria cannot be distinguished from mycobacterium tuberculosis *via* common diagnostic methods.

The main solution to fight with this disease is to identify the infected people and cure them *via* anti-tuberculosis drugs. This perspective is only feasible on these conditions, a consistent network of laboratories with an efficient and contemporary equipment and an instructed staff; and the other condition is a suitable managing system.

A laboratory is not limited to its building and equipments but it's a set of individuals and management systems that together ascertain processes and standards needed to achieve certain and quick results and then operate them. Accomplishing new diagnostic tests is possible with functional integrated systems, instructed and motivated staff, quality management systems and safe environment for working. Achieving this goal requires a precisely instructed staff, sufficient investment of organizations, individuals and government for equipment and laboratory management.

Despite of increasing sources of providing tools and equipments of diagnostic laboratories there are some deficiencies such as: insufficient fund, scant efforts to provide human resources for management and right leading of EQA, lack of suitable processes with the intention of generating and then performing quality systems, lack of practicable and logical standards, dearth of desired organizational structures and prerequisite conditions of laboratory services.

## CONCLUSION

 Instead of suggesting the technologic headways as the only diagnostic approach of tuberculosis, different organizations and countries with the intention of achieving the best diagnostic approach of tuberculosis have to share the same practical instructions around the world in order to enhance the management systems of laboratories immediately. So that the laboratory networks have to be considered as a complete system and then instructions can be communicated as technical guidelines and efficient guarantee of quality. The other prerequisite is: accordance and protection of different countries and organizations with the purpose of public education and supportive networks improvement by equipment enhancement.

## Figures and Tables

**Fig. (1):**
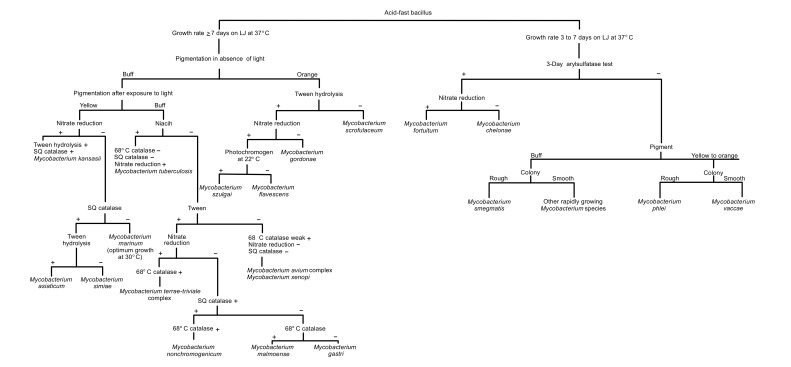
Schematic diagram for the identification of mycobacteria species.

**Table 1:** Biochemical properties of some clinically important mycobacteria.

**Species**	**Growth temp (°C)**	**Growth rate**	**Pigmentation**	Catalase (SQ^*^)	**Iron Uptake**	**Growth on McConkey**	**Tellurite Reduction**	**Arylsulfatase**		**Pyrazinamidase**	**Urease**	**Tween Hydrolysis**	**Nitrate Reduction**
								**2 weeks**	**3 days**				
*M. fortuitum*	22-40	R	N	98	99	96	97	99	97	-	93	50	99
*M. chelonae*	22-35	R	N	-97	-98	96	85	98	96	-	99	93	-99
*M. abscessus*	22-40	R	N	-97	-98	96	85	98	96	-	99	-47	-99
*M. ulcerans*	25-33	S	N	99	-	-	-	-	-	-	-	-	-
*M. tuberculosis*	33-39	S	N (99)	-99	-	-99	-70	-93	-99	98	98	68	99
*M. avium complex*	22-45	S (99%)	N	-99	-	-99	-70	-93	-99	99	99	99	99

* Semi Quantitative catalase ** Test reaction listed as ‘+’ or ‘-‘followed by the percentage strains reacting as indicated. If no percentage is given or space is blank, insufficient data were available or test was of no apparent value.

**Table 2:** Genes used for differentiation of mycobacterial species.

**Gene**	**Product**
***rrs***	16S rRNA
**ITS**	Internal transcribed spacer region
***hsp*65**	Heat shock protein 65
***gro*ES**	10-kDa chaperonin
***rec*A**	Recombination protein
***rpo*B**	DNA-directed RNA polymerase beta chain
***dna*J**	Chaperone protein
***oxy*R**	hydrogen peroxide-inducible gene activator
***pnc*A**	Pyrazinamidase/nicotinamidase
***rnp*B**	Catalytic subunit of RNase P
***sod*A**	Superoxide dismutase
***gyr*B**	DNA gyrase subunit B
***sec*A1**	Preprotein translocase

**Table 3:** Major genes of *M. tuberculosis* linked with acquisition of drug resistance.

**Drug**	**Gene**	**Product**
**Isoniazid**	*kat*G *inh*A *ndh* *ahp*C	Catalase-peroxidase-peroxynitritase T NADH-dependent enoyl-acyl carrier protein reductase NADH dehydrogenase Alkyl hydroperoxide reductase C
**Rifampin**	*rpo*B	DNA-directed RNA polymerase β chain
**Pyrazinamide**	*pnc*A	Pyrazinamidase/nicotinamidase
**Streptomycin**	*rps*L *rrs* *gid*B	30S ribosomal protein S12 16S rRNA Glucose-inhibited division protein B
**Amikacin-kanamycin**	*rrs* *eis*	16S rRNA Enhanced intracellular survival protein
**Ethionamide**	*eth*A *inh*A *eth*R *ndh*	Monooxygenase NADH-dependent enoyl-acyl carrier protein reductase TetR family transcriptional repressor NADH dehydrogenase
**Fluoroquinolones**	*gyr*AB	DNA gyrase
**para-Aminosalicylic acid**	*thy*A *fol*C	Thymidylate synthase Folylpolyglutamate synthase C

## LIST OF ABBREVIATIONS

BCG: = Bacillus Calmette–Guérin
TB: = Tuberculosis; NTMs: Non-tuberculous mycobacterium
HAIs: = hospital-acquired infections
MDR: = Multi Drugs Resistance
FM: = fluorescent microscopy
FIND: = Foundation for Innovative New Diagnostics
XDR: = Extensively drug-resistant
EQA: = External quality assessment
NAAT: = Nucleic acid amplification test
MIC: = Minimum Inhibitory Concentration
OADC: = Oleic Albumin Dextrose Catalase
MGIT: = Mycobacterial Growth Indicator Tube
DST: = Developmental systems theory
NRL: = National reference laboratories
